# Food sources of energy and nutrients in Finnish girls and boys 6–8 years of age – the PANIC study

**DOI:** 10.3402/fnr.v60.32444

**Published:** 2016-09-30

**Authors:** Aino-Maija Eloranta, Taisa Venäläinen, Sonja Soininen, Henna Jalkanen, Sanna Kiiskinen, Ursula Schwab, Timo A. Lakka, Virpi Lindi

**Affiliations:** 1 Department of Physiology, Institute of Biomedicine, School of Medicine, University of Eastern Finland, Kuopio, Finland; 2 Department of Clinical Nutrition, Institute of Public Health and Clinical Nutrition, School of Medicine, University of Eastern Finland, Kuopio, Finland; 3 Social and Health Center, City of Varkaus, Finland; 4 Institute of Dentistry, School of Medicine, University of Eastern Finland, Kuopio, Finland; 5 Department of Internal Medicine, Institute of Clinical Medicine, Kuopio University Hospital, Kuopio, Finland; 6 Kuopio Research Institute of Exercise Medicine, Kuopio, Finland; 7 Department of Clinical Physiology and Nuclear Medicine, Kuopio University Hospital, Kuopio, Finland

**Keywords:** food sources, energy, nutrients, vitamins, minerals, children, gender differences

## Abstract

**Background:**

Data on food sources of nutrients are needed to improve strategies to enhance nutrient intake among girls and boys in Western countries.

**Objective:**

To identify major food sources of energy, energy nutrients, dietary fibre, and micronutrients, and to study gender differences in these food sources among children.

**Design:**

We assessed food consumption and nutrient intake using 4-day food records in a population sample of Finnish girls (*n*=213) and boys (*n*=217) aged 6–8 years from the Physical Activity and Nutrition in Children Study. We calculated the percentual contribution of 55 food groups for energy and nutrient intake using the population proportion method.

**Results:**

Low-fibre grain products, skimmed milk, and high-fibre bread provided almost 23% of total energy intake. Skimmed milk was the top source of protein (18% of total intake), vitamin D (32%), potassium (20%), calcium (39%), magnesium (17%), and zinc (16%). Vegetable oils (15%) and high-fat vegetable oil–based spreads (14%) were the top sources of polyunsaturated fat. High-fibre bread was the top source of fibre (27%) and iron (12%). Non-root vegetables were the top source of folate (14%) and vitamin C (22%). Sugar-sweetened beverages provided 21% of sucrose intake. Pork was a more important source of protein and sausage was a more important source of total fat and monounsaturated fat in boys than in girls. Vegetable oils provided a higher proportion of unsaturated fat and vitamin E among boys, whereas high-fat vegetable oil–based spreads provided a higher proportion of these nutrients among girls.

**Conclusion:**

Commonly recommended foods, such as skimmed milk, high-fibre grain products, vegetables, vegetable oil, and vegetable oil–based spreads, were important sources of several nutrients, whereas sugar-sweetened beverages provided the majority of sucrose intake among children. This knowledge can be used in improving health among children by dietary interventions, nutrition education, and health policy decision making.

Children in Western countries fail to meet recommendations for many nutrients that are important for their health, growth, and development (1). For example, the intakes of saturated fat (SFA), sucrose, and salt are higher and the intakes of vitamin D, iron, and dietary fibre as well as unsaturated-to-saturated fatty acid ratio are lower than recommended among children in many Western countries, including Finland (2, 3).

Health promotion messages and nutrition counselling can be made more concrete and easier to adopt if they are based on food consumption rather than nutrient intake. Therefore, recent dietary guidelines, for example in Nordic countries, emphasise healthy dietary choices at food rather than nutrient level (4, 5). Consequently, it is important to have precise data on food sources of nutrients to better design strategies to enhance diet quality in different age groups.

There are few studies on the food sources of nutrients in children and most of them have reported the sources of only one or few nutrients (6–9). Milk products have been found to be among the main sources of energy, protein, and SFA among children in Europe (6, 8, 10) and the USA (11–13). Sugar-sweetened beverages have also been observed to be a major source of energy and refined carbohydrates among US children (13, 14). In a Swedish study, girls obtained more sucrose from sweets and chocolate, whereas boys received more sucrose from sugar-sweetened beverages (8). With the exception of the Swedish study (8), there are limited data on differences in the food sources of nutrients among girls and boys (11).

To provide detailed information on food sources of nutrients for health promotion and dietary counselling to correct the observed shortcomings in nutrient intake among children, we investigated the main food sources of energy, energy nutrients, dietary fibre, vitamins, and minerals among Finnish children 6–8 years of age. We also studied the gender differences in food sources of energy and these nutrients.

## Methods

### Study population

The present analyses are based on the baseline data of the Physical Activity and Nutrition in Children (PANIC) study, which is an ongoing physical activity and dietary intervention study in a population sample of primary school children from the city of Kuopio, Finland. We invited 736 children 6–8 years of age who were registered for the first grade in 16 primary schools, selected out of all 26 primary schools of Kuopio, in 2007–2009. We received the contact information of the children’s principal custodians from the city of Kuopio and sent them the invitation letters by mail. Of 736 invited children, 512 (70%) participated in the baseline examinations that were conducted in 2007–2009. The participants did not differ in sex distribution, age, or body mass index standard deviation score (BMI-SDS) from all children who started first grade in primary schools of Kuopio in 2007–2009 based on available school health examination data. Complete dietary data were available for 430 children (213 girls and 217 boys) who were included in the analyses. These children did not differ in sex distribution, age, or BMI-SDS from those 82 children who had incomplete data and were excluded. The PANIC study protocol was approved by the research ethics committee of the Hospital District of Northern Savo. All participating children and their parents gave informed written consent.

### Assessments

We assessed food consumption and nutrient intake by food records administered by the parents on 4 predefined consecutive days that included either consecutive 2 weekdays and 2 weekend days (99.5%) or consecutive 3 weekdays and 1 weekend day (0.5%). A clinical nutritionist instructed the parents to record all food and drinks consumed by their child at home, at school, in afternoon care, and elsewhere outside home using household or other measures, such as tablespoons, decilitres, and centimetres, in person at the first study visit. The parents were instructed to report the recipes of mixed dishes and the brands and the contents of food products. A clinical nutritionist reviewed the food records with the parents at the second study visit and completed the records using a picture booklet of portion sizes (15). Moreover, a clinical nutritionist asked the catering company about the details of food and drinks, such as menus, cooking fat, and spread on bread, served at schools and in afternoon care. All prepared foods and mixed dishes were disaggregated into ingredients according to the recipes used. We analysed food consumption and nutrient intake using The Micro Nutrica^®^ dietary analysis software, version 2.5 (The Social Insurance Institution of Finland), that uses Finnish and international data on the nutrient compositions of foods (16). A clinical nutritionist also updated the software by adding new food items and products with their precise nutrient content received from the producers. Vitamin and mineral supplements were not included in these analyses. Food records that contained <4 days, contained inconsecutive days, did not include weekdays and weekend days, or lacked essential information for dietary analysis even after the review were excluded from the analyses. We estimate that 26% of girls and 24% of boys may have underreported their total energy intake in the food records when comparing it with energy expenditure estimated by basal metabolic rate and using the cut-offs for underreporting suggested by Torun et al. (17).

Foods consumed were divided into 55 food groups each of which included foods of similar nutrient composition and type of consumption (Table 1). We divided bread, breakfast cereals, and grain products into six food groups based on their fibre content. Bread, breakfast cereals, and grain products with at least 5% of fibre were considered high-fibre products. Meat products were divided into seven groups and fish was separated from shellfish and fish products. Fats and fat products were divided into five groups based on the amount and the quality of fat. Milk, sour milk products, and cheese were divided into six groups based on their fat content. Milk and sour milk products with <1% of fat were considered low-fat products. Cheese was defined low-fat cheese if it contained no more than 17% of fat. Ice creams and puddings were included in the same group. Sweetened beverages were divided into artificially sweetened and sugar-sweetened beverages. Energy drinks formed one group.

**Table 1:** Contents and median intakes (interquartile range) of the food groups in girls and boys

Food groups	Contents	All children (*n*=430), median (interquartile range)	Girls (*n*=213), median (interquartile range)	Boys (*n*=217), median (interquartile range)
High-fibre bread	Rye bread, crisp bread, wholegrain bread (fibre ≥5%)	37.5 (22.5, 59.0)	36.5 (21.1, 58.9)	37.5 (22.5, 59.0)
Low-fibre bread	White bread, white rolls (fibre <5%)	22.0 (7.5, 40.1)	22.3 (7.5, 37.5)	21.7 (7.5, 45.0)
High-fibre breakfast cereals	Mueslis, breakfast cereals (fibre ≥5%)	0.0 (0.0, 3.8)	0.0 (0.0, 3.8)	0.0 (0.0, 4.1)
Low-fibre breakfast cereals	Breakfast cereals (fibre <5%)	0.0 (0.0, 7.5)	0.0 (0.0, 7.5)	0.0 (0.0, 6.6)
High-fibre grain products	Wholegrain pasta, rice, oatmeal (fibre ≥5%)	9.6 (0.0, 24.1)	9.4 (0.0, 23.5)	10.5 (0.0, 24.9)
Low-fibre grain products	White pasta, rice, flours (fibre <5%)	70.7 (45.0, 104.7)	71.8 (46.2, 100.4)	69.1 (42.6, 108.5)
Sweet pastry	Biscuits, cookies, cakes, pastries	0.0 (0.0, 6.8)	0.0 (0.0, 6.6)	0.0 (0.0, 7.1)
Potato	Potatoes	70.7 (44.7, 101.2)	68.3 (43.6, 100.4)	75.0 (48.3, 101.9)
Root vegetables	Carrot, beetroot, turnip	14.9 (3.8, 30.2)	16.9 (5.3, 31.6)	12.5 (1.7, 28.2)
Non-root vegetables	Green leafy vegetables, cabbages, cucumber, peppers, tomatoes, onions	68.3 (40.0, 103.9)	68.3 (43.1, 103.9)	68.3 (39.0, 104.8)
Legume and nut	Peas, beans, nuts, almonds, seeds	0.0 (0.0, 2.4)	0.0 (0.0, 2.2)	0.0 (0.0, 2.5)
Mushroom	Mushrooms	0.0 (0.0, 0.0)	0.0 (0.0, 0.0)	0.0 (0.0, 0.0)
Vegetable products	Ready-made vegetable products	0.0 (0.0, 0.0)	0.0 (0.0, 0.0)	0.0 (0.0, 0.0)
Fruit	Fruit and fruit purées	73.8 (33.6, 121.1)	75.8 (40.5, 126.8)	70.0 (27.5, 117.7)
Berry	Berries and berry purées	9.0 (0.0, 25.4)	9.9 (0.0, 28.2)	8.3 (0.0, 23.4)
Jam	Jams, marmalades	0.9 (0.0, 7.7)	1.6 (0.0, 8.0)	0.0 (0.0, 7.4)
Pork	Pork	29.5 (15.7, 46.8)	27.8 (13.8, 42.6)	32.6 (16.7, 56.5)**
Beef	Beef	13.5 (0.0, 29.0)	12.0 (0.0, 27.9)	14.5 (0.0, 30.5)
Sausage	Frankfurter, balkan, salami, ham sausage	15.0 (3.5, 31.8)	11.8 (0.0, 25.3)	18.0 (6.9, 34.6)**
Poultry	Chicken, turkey	10.3 (0.0, 24.5)	10.3 (0.0, 22.9)	10.0 (0.0, 27.2)
Game	Hare, moose, willow grouse	0.0 (0.0, 0.0)	0.0 (0.0, 0.0)	0.0 (0.0, 0.0)
Lamb and other meat	Lamb, horse, reindeer	0.0 (0.0, 0.0)	0.0 (0.0, 0.0)	0.0 (0.0, 0.0)
Organ meats	Liver, kidney, tongue	0.0 (0.0, 0.0)	0.0 (0.0, 0.0)	0.0 (0.0, 0.0)
Fish	Fresh fish	0.0 (0.0, 18.5)	0.0 (0.0, 17.1)	0.0 (0.0, 23.9)
Shellfish	Shellfish	0.0 (0.0, 0.0)	0.0 (0.0, 0.0)	0.0 (0.0, 0.0)
Fish products	Salted fish, smoked fish, canned fish, roe paste	0.0 (0.0, 0.0)	0.0 (0.0, 0.0)	0.0 (0.0, 0.0)
Egg	Eggs	10.0 (5.7, 19.1)	9.8 (5.8, 18.6)	10.9 (5.6, 19.6)
High-fat vegetable oil–based spreads	Vegetable oil–based spreads (fat 60–80%)^a^	5.0 (1.3, 10.3)	5.0 (1.5, 10.6)	4.5 (1.3, 9.9)
Low-fat vegetable oil–based spreads	Low-fat vegetable oil–based spreads (fat <60%)^a^	0.0 (0.0, 5.3)	0.0 (0.0, 5.0)	0.0 (0.0, 6.1)
Vegetable oil	Vegetable oils, vegetable oil–based salad dressings	2.9 (1.2, 5.5)	2.5 (1.1, 5.0)	3.1 (1.2, 6.3)
Butter and butter-based spreads	Butter, butter-based spreads	3.0 (0.9, 7.9)	2.9 (1.0, 6.9)	3.1 (0.8, 10.1)
Shortenings and other added fats	Shortenings, lard, coconut fat	5.1 (2.7, 8.5)	5.1 (2.7, 8.6)	5.2 (2.7, 8.6)
Skimmed milk	Milk,^a^ organic milk (fat <1%)	371.7 (88.8, 602.8)	356.4 (91.4, 587.5)	421.2 (87.5, 633.8)
Fat-containing milk	Milk,^a^ organic milk, farm milk (fat ≥1%)	95.8 (33.1, 264.4)	93.4 (31.2, 253.7)	102.6 (36.6, 301.7)
Low-fat sour milk products	Yoghurts, sour milk (fat <1%)	0.0 (0.0, 9.9)	0.0 (0.0, 13.3)	0.0 (0.0, 8.9)
Sour milk products	Yoghurts, sour milk (fat ≥1%)	66.9 (25.0, 125.0)	65.0 (25.0, 114.4)	75.0 (25.0, 142.6)
Low-fat cheese	Cheese, cottage cheese, processed cheese, fromage frais (fat ≤17%)	2.5 (0.0, 10.0)	4.2 (0.0, 12.0)	0.6 (0.0, 9.0)*
Cheese	Cheese, processed cheese, blue cheese, fromage frais (fat >17%)	4.2 (0.0, 10.1)	3.8 (0.0, 9.3)	5.0 (0.0, 11.2)
Ice cream and pudding	Ice creams, puddings	17.5 (0.0, 37.9)	16.3 (0.0, 40.9)	18.8 (0.0, 37.5)
Cream and other dairy products	Cream, sour cream, double cream, soya-, rice-, and oat-based milks and creams	10.6 (2.9, 20.0)	11.2 (3.8, 19.9)	10.4 (1.6, 20.2)
Coffee	Coffee	0.0 (0.0, 0.0)	0.0 (0.0, 0.0)	0.0 (0.0, 0.0)
Tea	Tea	0.0 (0.0, 0.0)	0.0 (0.0, 0.0)	0.0 (0.0, 0.0)
Artificially sweetened beverage	Carbonated and non-carbonated artificially sweetened beverages	0.0 (0.0, 50.0)	0.0 (0.0, 0.0)	0.0 (0.0, 56.7)
Sugar-sweetened beverage	Carbonated and non-carbonated sugar-sweetened beverages	100.0 (43.4, 200.3)	87.5 (42.5, 200.0)	125.0 (50.0, 225.0)
Fruit juice	Natural fruit juices	1.4 (0.0, 50.0)	1.5 (0.0, 50.0)	0.8 (0.0, 50.0)
Energy drink	Energy drinks	0.0 (0.0, 0.0)	0.0 (0.0, 0.0)	0.0 (0.0, 0.0)
Water and other drinks	Water, mineral water, sport drink, mulled wine, berry fool	187.2 (108.2, 319.8)	163.4 (90.1, 277.8)	206.0 (121.6, 350.0)**
Sugar and honey	Table sugar, baking sugar, honey, syrup	7.7 (4.2, 13.3)	7.7 (4.1, 13.3)	7.4 (4.4, 13.3)
Candy	Sweets, chewing gums	12.5 (2.0, 31.1)	12.0 (2.0, 26.8)	15.0 (0.0, 37.5)
Chocolate and hot chocolate powder	Chocolate, hot chocolate powder	5.3 (0.2, 14.1)	5.0 (0.5, 12.5)	6.0 (0.0, 16.2)
Ready meal	Hamburgers, pizzas, nuggets	0.0 (0.0, 10.0)	0.0 (0.0, 5.6)	0.0 (0.0, 15.0)
Salt	Recipe and table salt	2.2 (1.8, 2.8)	2.1 (1.7, 2.6)	2.4 (1.9, 3.0)***
Condiments and sauces	Ketchup, mustard, soy sauce, chilli sauce, barbeque sauce	3.9 (0.4, 12.0)	4.0 (0.5, 13.0)	3.8 (0.3, 11.1)
Salty snacks	Popcorn, potato crisps, nachos, crackers, tacos	0.0 (0.0, 3.8)	0.0 (0.0, 3.8)	0.0 (0.0, 3.9)

a Fortified with vitamin D.

* *p*<0.05 for difference between girls and boys.

** *p*<0.01 for difference between girls and boys.

*** *p*<0.001 for difference between girls and boys.

### Statistical methods

Data were analysed using the SPSS for Windows software, Version 21.0 (IBM Corp. IBM SPSS Statistics for Windows, Armonk, NY). We compared differences in food consumption between genders by the Mann–Whitney U-test. The differences between genders with *p*-value <0.05 were considered statistically significant. We calculated the contribution of each food group to the intakes of total energy, energy nutrients, dietary fibre, and selected vitamins and minerals using the population proportion method as defined by Krebs-Smith et al. (18). The percentage contribution of each food group to the intakes of total energy, energy nutrients, dietary fibre, and selected vitamins and minerals was estimated by summing the amount of a particular nutrient from the particular food group for all children and dividing this value by the sum of the nutrient from all foods for all children. The food sources of nutrients were ranked and top 10 sources were reported for all children, and for girls and boys separately.

## Results

The medians (interquartile ranges) of the consumption of food groups in girls and boys are presented in Table 1. Boys consumed more pork, sausage, water and other drinks, and salt and less low-fat cheese than girls.

### Food sources of energy, energy nutrients, and dietary fibre

Several food groups contributed almost similarly to the intake of energy, the two main sources being low-fibre grain products and skimmed milk in girls and boys (Table 2). Skimmed milk was the main source of protein in girls and boys, followed by fat-containing milk in girls and pork in boys. The main source of total fat was high-fat vegetable oil–based spreads, followed by shortenings and other added fats among girls and sausage among boys. The top two sources of SFA were cream and other dairy products and fat-containing milk in girls and fat-containing milk and butter and butter-based spreads in boys. The main source of monounsaturated fatty acids (MUFA) was high-fat vegetable oil–based spreads in girls and boys, followed by shortenings and other added fats among girls and by sausage among boys. The main source of polyunsaturated fatty acids (PUFA) was high-fat vegetable oil–based spreads followed by vegetable oil in girls and vegetable oil followed by high-fat vegetable oil–based spreads in boys. Low-fibre grain products were the major source of carbohydrates, followed by skimmed milk in girls and boys. Sugar-sweetened beverages and sugar and honey were the major sources of sucrose in both girls and boys. High-fibre bread was the major source of dietary fibre, followed by fruit and low-fibre grain products in girls and boys.

**Table 2:** Dietary sources of energy, energy nutrients, and dietary fibre in all children, girls, and boys

		All (*n*=430)	Girls (*n*=213)	Boys (*n*=217)
				
Rank^a^	Food group	% of intake	% of intake	% of intake
Energy				
1	Low-fibre grain products	9.4	9.5	9.4
2	Skimmed milk	7.6	7.5	7.7
3	High-fibre bread	5.5	5.7	5.4
4	Fat-containing milk	5.4	5.4	5.3
5	Sour milk products	4.5	4.3	4.6
6	Candy	4.4	4.3	4.6
7	Low-fibre bread	4.0	4.0	4.0
8	Pork	3.7	3.4	3.9
9	Sugar-sweetened beverage	3.3	3.2	3.4
10	Potato	3.3	3.3	3.2
Protein				
1	Skimmed milk	18.3	18.2	18.4
2	Pork	9.2	8.7	9.7
3	Fat-containing milk	9.2	9.4	9.0
4	Low-fibre grain products	6.5	6.6	6.4
5	Beef	5.8	5.9	5.7
6	High-fibre bread	5.0	5.1	4.8
7	Poultry	4.6	4.8	4.4
8	Sour milk products	4.3	4.2	4.3
9	Sausage	3.7	3.1	4.2
10	Low-fibre bread	3.6	3.6	3.6
Total fat				
1	High-fat vegetable oil–based spreads	9.1	10.0	8.4
2	Shortenings and other added fats	8.1	8.6	7.7
3	Sausage	7.2	6.2	8.0
4	Pork	6.9	6.5	7.2
5	Butter and butter-based spreads	6.6	6.3	6.8
6	Vegetable oil	6.5	6.2	6.8
7	Fat-containing milk	5.4	5.3	5.4
8	Cream and other dairy products	5.3	5.8	4.8
9	Chocolate and hot chocolate powder	5.0	4.6	5.3
10	Ice cream and pudding	4.8	5.3	4.4
Saturated fatty acids				
1	Fat-containing milk	8.8	8.7	8.9
2	Cream and other dairy products	8.3	9.2	7.5
3	Butter and butter-based spreads	7.9	7.7	8.1
4	Shortenings and other added fats	7.9	8.3	7.5
5	Ice cream and pudding	7.2	7.9	6.6
6	Chocolate and hot chocolate powder	7.0	6.5	7.4
7	Sausage	6.8	5.8	7.6
8	High-fat vegetable oil–based spreads	6.6	7.3	6.0
9	Pork	6.2	5.9	6.5
10	Cheese	6.2	5.5	6.8
Monounsaturated fatty acids				
1	Vegetable oil–based spreads	12.2	13.4	11.2
2	Sausage	9.6	8.3	10.7
3	Vegetable oil	9.3	8.9	9.6
4	Pork	9.0	8.4	9.4
5	Shortenings and other added fats	8.5	9.0	8.1
6	Butter and butter-based spreads	5.3	5.0	5.7
7	Chocolate and hot chocolate powder	4.8	4.4	5.0
8	Cream and other dairy products	4.0	4.3	3.8
9	Ice cream and pudding	3.5	3.8	3.2
10	Fat-containing milk	3.2	3.2	3.3
Polyunsaturated fatty acids				
1	Vegetable oil	15.0	14.1	15.8
2	High-fat vegetable oil–based spreads	13.8	14.8	12.9
3	Shortenings and other added fats	11.0	11.9	10.2
4	Sausage	5.9	5.1	6.6
5	Pork	5.9	5.5	6.2
6	Low-fat vegetable oil–based spreads	5.5	4.9	6.0
7	Salty snacks	5.5	5.7	5.2
8	Low-fibre grain products	4.2	4.2	4.1
9	High-fibre bread	3.4	3.5	3.3
10	Butter and butter-based spreads	3.2	2.8	3.5
Carbohydrates				
1	Low-fibre grain products	14.8	14.8	14.8
2	Skimmed milk	8.5	8.3	8.7
3	Candy	8.3	7.9	8.7
4	High-fibre bread	8.0	8.2	7.9
5	Sugar-sweetened beverage	6.1	5.9	6.2
6	Low-fibre bread	5.7	5.6	5.7
7	Potato	5.5	5.6	5.4
8	Sour milk products	5.2	4.9	5.4
9	Fruit	5.0	5.5	4.5
10	Sugar and honey	4.5	4.7	4.3
Sucrose				
1	Sugar-sweetened beverage	21.2	20.5	21.7
2	Sugar and honey	17.5	18.4	16.6
3	Sour milk products	14.5	13.6	15.3
4	Candy	9.7	9.2	10.1
5	Chocolate and hot chocolate powder	8.0	7.4	8.5
6	Ice cream and pudding	6.4	6.8	6.0
7	Fruit	6.2	6.8	5.6
8	Sweet pastry	2.4	2.2	2.6
9	Fruit juice	2.4	2.1	2.6
10	Low-fibre breakfast cereals	2.0	2.2	1.9
Dietary fibre				
1	High-fibre bread	27.1	27.1	27.2
2	Fruit	11.7	12.4	11.0
3	Low-fibre grain products	10.9	10.7	11.0
4	High-fibre grain products	8.3	7.7	8.7
5	Potato	8.1	8.1	8.1
6	Low-fibre bread	7.5	7.1	7.8
7	Non-root vegetables	6.0	6.3	5.6
8	Berry	3.6	3.7	3.5
9	Root vegetables	3.3	3.7	3.0
10	High-fibre breakfast cereals	2.0	1.8	2.2

a Rank for all children.

### Food sources of vitamins

Skimmed milk and fat-containing milk were the most important sources of vitamin D, followed by high-fat vegetable oil–based spreads among girls and by fish among boys (Table 3). The main source of vitamin E was high-fat vegetable oil–based spreads followed by vegetable oil in girls and vegetable oil followed by high-fat vegetable oil–based spreads in boys. The main sources of folate were non-root vegetables and potato in girls and boys. Non-root vegetables were the main source of vitamin C, followed by sugar-sweetened beverages among girls and boys.

**Table 3:** Dietary sources of selected vitamins in all children, girls, and boys

		All (*n*=430)	Girls (*n*=213)	Boys (*n*=217)
				
Rank^a^	Food group	% of intake	% of intake	% of intake
Vitamin D				
1	Skimmed milk^b^	31.9	32.0	31.8
2	Fat-containing milk^b^	16.1	16.7	15.6
3	High-fat vegetable oil–based spreads^b^	11.1	12.2	10.1
4	Fish	9.7	8.4	10.8
5	Low-fat vegetable oil–based spreads^b^	6.5	6.0	6.9
6	Shortenings and other added fats	6.3	6.7	6.0
7	Fish products	3.0	2.8	3.1
8	Egg	2.9	3.1	2.7
9	Pork	2.5	2.4	2.6
10	Butter and butter-based spreads	1.5	1.3	1.7
Vitamin E				
1	Vegetable oil	14.1	13.1	15.1
2	High-fat vegetable oil-based spreads	12.8	13.5	12.2
3	Non-root vegetables	5.4	5.8	5.0
4	High-fibre bread	5.2	5.4	5.1
5	Low-fat vegetable oil–based spreads	5.1	4.4	5.6
6	Shortenings and other added fats	4.4	4.4	4.4
7	Fruit	4.1	4.6	3.7
8	Low-fibre bread	3.9	4.0	3.8
9	Egg	3.8	3.9	3.6
10	Butter and butter-based spreads	3.1	2.7	3.4
Folate				
1	Non-root vegetables	13.5	14.4	12.7
2	Potato	11.5	11.5	11.5
3	Skimmed milk	9.3	8.9	9.7
4	Sour milk products	6.5	6.2	6.8
5	High-fibre bread	6.0	6.2	5.8
6	Fruit	5.8	6.3	5.3
7	Fat-containing milk	4.5	4.4	4.6
8	Low-fibre grain products	3.9	3.7	4.0
9	Low-fibre bread	3.8	3.6	4.0
10	Egg	3.3	3.3	3.3
Vitamin C				
1	Non-root vegetables	22.3	23.1	21.4
2	Sugar-sweetened beverage	18.4	17.3	19.5
3	Fruit	14.4	15.9	12.9
4	Fruit juice	13.6	12.6	14.5
5	Berry	10.5	10.3	10.7
6	Potato	7.2	7.0	7.3
7	Skimmed milk	4.9	4.6	5.2
8	Fat-containing milk	2.4	2.3	2.5
9	Root vegetables	2.0	2.1	2.0
10	Sour milk products	0.9	0.8	1.0

a Rank for all children.

b Fortified with vitamin D.

### Food sources of minerals

Sodium was mainly obtained from salt among girls and boys (Table 4). The main source of potassium was skimmed milk, followed by potato in girls and fat-containing milk in boys. Skimmed milk and fat-containing milk provided the majority of calcium intake in girls and boys. High-fibre bread was the main source of iron, followed by low-fibre grain products in girls and boys. The main sources of magnesium were skimmed milk and high-fibre bread in girls and boys. Similarly, skimmed milk was the main source of zinc, followed by high-fibre bread among girls and boys.

**Table 4:** Dietary sources of selected minerals in all children, girls, and boys

		All (*n*=430)	Girls (*n*=213)	Boys (*n*=217)
				
Rank^a^	Food group	% of intake	% of intake	% of intake
Sodium				
1	Salt	37.8	37.9	37.7
2	High-fibre bread	8.2	8.6	7.9
3	Skimmed milk	6.9	6.9	6.9
4	Sausage	6.6	5.8	7.3
5	Low-fibre bread	4.8	4.8	4.8
6	Pork	4.1	3.7	4.4
7	Fat-containing milk	3.2	3.3	3.1
8	Condiments and sauces	3.2	3.4	3.0
9	Ready meal	2.1	1.9	2.2
10	Low-fibre breakfast cereals	1.8	1.9	1.7
Potassium				
1	Skimmed milk	20.4	19.9	20.9
2	Potato	9.7	9.9	9.5
3	Fat-containing milk	9.7	9.7	9.6
4	Non-root vegetables	6.9	7.3	6.6
5	Fruit	6.3	6.8	5.8
6	Sour milk products	5.4	5.1	5.6
7	High-fibre bread	5.0	5.0	5.0
8	Sugar-sweetened beverages	2.9	2.8	3.0
9	Low-fibre grain products	2.8	2.8	2.8
10	Pork	2.8	2.6	3.0
Calcium				
1	Skimmed milk	38.9	38.6	39.2
2	Fat-containing milk	19.5	19.9	19.2
3	Sour milk products	8.6	8.3	8.8
4	Cheese	5.3	4.6	5.9
5	Low-fat cheese	4.8	5.3	4.4
6	Ice cream and pudding	2.5	2.7	2.4
7	Chocolate and hot chocolate powder	1.9	1.7	2.0
8	Low-fat sour milk products	1.9	1.9	1.8
9	Candy	1.6	1.5	1.6
10	Non-root vegetables	1.5	1.6	1.4
Iron				
1	High-fibre bread	12.4	12.7	12.0
2	Low-fibre grain products	9.3	9.4	9.2
3	Potato	6.2	6.3	6.0
4	Low-fibre bread	5.9	5.8	6.0
5	High-fibre grain products	5.9	5.7	6.1
6	Pork	5.4	5.1	5.6
7	Beef	4.2	4.3	4.2
8	Egg	3.7	3.9	3.6
9	Candy	3.7	3.5	3.9
10	Non-root vegetables	3.6	3.9	3.4
Magnesium				
1	Skimmed milk	16.7	16.4	17.1
2	High-fibre bread	11.7	11.9	11.6
3	Fat-containing milk	7.7	7.8	7.7
4	Potato	6.6	6.6	6.5
5	Fruit	5.0	5.4	4.6
6	Low-fibre grain products	4.8	4.9	4.7
7	High-fibre grain products	4.6	4.4	4.7
8	Low-fibre bread	4.3	4.2	4.4
9	Non-root vegetables	4.2	4.5	4.0
10	Sour milk products	3.9	3.7	4.1
Zinc				
1	Skimmed milk	16.2	16.1	16.3
2	High-fibre bread	9.8	10.1	9.5
3	Pork	9.0	8.6	9.4
4	Fat-containing milk	7.8	8.0	7.7
5	Beef	7.4	7.6	7.3
6	Low-fibre grain products	4.9	5.0	4.7
7	Low-fibre bread	4.0	3.9	4.0
8	Sour milk products	3.8	3.7	3.9
9	High-fibre grain products	3.8	3.7	4.0
10	Sausage	3.4	2.9	3.9

a Rank for all children.

## Discussion

Our study in a population sample of girls and boys 6–8 years of age provides new information on the top food sources of energy, energy nutrients, dietary fibre, and several vitamins and minerals among primary school children. Previous studies have mainly focused on the sources of one or few nutrients (9, 19–21), and only few studies have compared the food sources of nutrients among girls and boys (8, 11).

Milk products have been observed to be among the main sources of energy, protein, and SFA among children in Western countries (6, 8, 10–13). However, most of the previous studies have not analysed the contribution of milk products of different fat contents to the intake of nutrients. We found that skimmed milk was among the top sources of energy, protein, carbohydrates, vitamin D, folate, potassium, calcium, magnesium, and zinc. Fat-containing milk provided a lower proportion of all these nutrients than skimmed milk. This finding is due to the higher consumption of skimmed milk than fat-containing milk in the present study sample that is in line with the results of previous studies in Finnish children (1, 22). However, we observed that fat-containing milk was the highest source of SFA among children. Since milk is commonly consumed in large quantities among children, the consumption of fat-containing milk easily leads to an excessive intake of SFA. Because milk is an important source of several nutrients among children, sufficient intake of these nutrients from other sources should be assured among children who do not drink milk regularly.

Bread has been reported to provide 20–45% of the intake of dietary fibre among Finnish children (6) and European adolescents (23). We found that high-fibre bread was among the top sources not only for dietary fibre but also for iron, magnesium, and zinc. However, low-fibre grain products were the top source of energy and carbohydrates among children. These findings are explained by a higher density of dietary fibre and minerals but a lower consumption of high-fibre grain products than those of low-fibre grain products. Enhancing the use of high-fibre grain products at the expense of low-fibre grain products could be effective in improving the intake of several nutrients among children.

Previous studies have reported that sugar-sweetened beverages provide a considerable amount of energy and refined carbohydrates among children (8, 13, 14). In line with those findings, sugar-sweetened beverages were the top source of sucrose in children in this study, providing one-fifth of total sucrose intake. They were also the ninth most common source of energy and the fifth most common source of carbohydrates. The reduction in the consumption of sugar-sweetened beverages among children would markedly decrease their total intake of sucrose, which is currently higher than recommended (1, 24). Moreover, we found that sugar and honey provided almost 18% of the total sucrose intake in children. In this study, the food group of sugar and honey included all sugar used at home in baking, cooking, and as table sugar, and the dietary analysis software was unable to analyse sugar from recipes and sugar as such separately. This led to overestimation of sugar and honey as a source of sucrose at the expense of other food groups and needs to be acknowledged when comparing the dietary sources of sucrose in this study with those of other studies.

Previous studies have shown that the daily intake of vitamin D from food is around 6 µg in Finland (24–26) and even lower in the most Western countries (2, 3, 27). Products fortified with vitamin D have been reported to be major food sources of vitamin D in children across Europe and USA (10, 26–28). Whereas products fortified with vitamin D are available to a lesser extent in many other countries, in Finland most of the liquid dairy products and vegetable oil-based spreads have been fortified with vitamin D since 2003. In line with this, we found that the top sources of vitamin D among children were skimmed milk, fat-containing milk, and high-fat vegetable oil–based spreads that together contributed more than a half of the total intake of vitamin D. The level of fortification was increased in 2010, after the data collection of this study, in both liquid dairy products and fat spreads. Therefore, we suggest that the fortified products are even more abundant sources of vitamin D in Finnish children at the moment. Moreover, because skimmed milk was among the top sources of several vitamins and minerals, and high-fat vegetable oil–based spreads were among the top sources of MUFA, PUFA, and vitamin E, these products are not only good sources of vitamin D but also important sources of other nutrients among children.

Potato was among the top 10 sources of energy, carbohydrates, dietary fibre, folate, vitamin C, potassium, iron, and magnesium in our study sample. Although potato is not particularly rich in these nutrients (16, 29), high consumption of potato in Finland makes it as a good source of several nutrients. On the contrary, although the consumption of vegetables and fruit was relatively low among children in this study, they contributed markedly to the intakes of dietary fibre, vitamin E, folate, and vitamin C and to a lesser extent to the intakes of several minerals. This is due to the high density of these nutrients in vegetables and fruit (29).

Fish and fish products were among the top 10 sources of vitamin D in our study. However, fish did not markedly contribute to the intakes of any other nutrients, although it is high in protein and several minerals, such as calcium and potassium, and some fish species provide a considerable amount of PUFA (29). This finding is explained by the low consumption of fish among children in our study, which is in line with a previous report among Finnish children (1, 24).

In one previous study, meat provided more total and SFA in boys than in girls among 15-year-old adolescents, but the differences between genders were smaller among 9-year-old children (8). We also found that meat and sausages covered a slightly higher proportion of the intake of protein, fat, and sodium in boys than in girls. Moreover, vegetable oils provided a higher proportion of PUFA and vitamin E among boys, whereas high-fat vegetable oil–based spreads provided a higher proportion of these nutrients among girls. However, the rankings of most sources of nutrients did not differ between girls and boys. One explanation for the minor differences between girls and boys can be that our study sample consisted of primary school children who are provided a free-of-charge school meal every school day which may equalise food consumption between genders at this age. In addition, most of the gender differences in the sources of nutrients may not appear before adulthood (30).

A major strength of this study is the large population-based sample of Finnish primary school children. Another strength is that food consumption and nutrient intake were assessed by 4-day food records that were reviewed by a clinical nutritionist together with the family at return and analysed using a carefully updated nutrition database. A limitation of this study is that the grouping of food items was based on the judgement of a clinical nutritionist which could have had an influence on the rankings. If no detailed information was available, standard recipes were used for foods prepared at home, which could have led to either underestimation or overestimation of the intake of certain nutrients at an individual level. However, at group level this might have a minor effect only.

This study provides comprehensive data on the main food sources of nutrients among primary school girls and boys. Foods that are commonly recommended to be included in a healthy diet, such as skimmed milk, high-fibre grain products, vegetables, vegetable oil, and vegetable oil-based spreads, had a major contribution to the intake of several health-promoting nutrients, whereas sugar-sweetened beverages provided the majority of sucrose intake. This knowledge can be used in improving health among children by dietary interventions, nutrition education, and health policy decision making.
